# Exploring factors influencing digitally disadvantaged students' behavioral intention to use educational television programs

**DOI:** 10.3389/fpsyg.2026.1729219

**Published:** 2026-07-17

**Authors:** Hetiao Hong, Xuwen Dong, Xinyuan Chen, Zhangqin Wang, Wenjuan Zhu

**Affiliations:** 1 Chinese Education Modernization Research Institute, Hangzhou Normal University (Zhejiang Provincial Key Think Tank), Hangzhou, Zhejiang, China; 2 Jing Hengyi School of Education, Hangzhou Normal University, Hangzhou, Zhejiang, China; 3 Wasu Media Network Co., Ltd, Hangzhou, Zhejiang, China; 4 Educational Technology Center of Zhejiang Province, Zhejiang, China

**Keywords:** behavioral intention, digitally disadvantaged students, educational television programs, PLS-SEM, UTAUT2

## Abstract

**Introduction:**

Educational television programs serve as a potentially accessible learning medium for digitally disadvantaged students, yet the factors shaping their behavioral intention to use such programs remain underexplored. Grounded in the Unified Theory of Technology Acceptance and Use 2 (UTAUT2), this study investigates what drives digitally disadvantaged students to form intentions around using educational television programming.

**Methods:**

Partial least squares structural equation modeling (PLS-SEM) was employed as the analytical approach to examine the relationships among seven UTAUT2 constructs and behavioral intention.

**Results:**

Three constructs-Hedonic Motivation (HM), Habit (H), and Facilitating Conditions (FC)-emerged as meaningful predictors of behavioral intention (BI), with HM being the strongest predictor. Social Influence (SI), Effort Expectancy (EE), Performance Expectancy (PE), and Price Value (PV) failed to reach statistical significance. The model's explanatory capacity for behavioral intention was moderate (*R*^2^ = 0.347).

**Discussion:**

For digitally disadvantaged students, the intention to use educational television programs is more influenced by media experience, daily contact, and actual support conditions than by instrumental evaluation, social norms, or price considerations. Beyond extending UTAUT2 to the domain of televised instruction and digitally constrained populations, this investigation yields practical pointers for designing and sustaining accessible learning media.

## 1 Introduction

In recent years, educational television programs have received renewed attention in many countries and regions during the COVID-19 outbreak, when school closures and the breakdown of in-person teaching led numerous education systems to reintroduce television- and radio-based educational provision as part of emergency remote learning strategies (Asis and Bayani, 2023; Ayanwale et al., 2023; Mateus et al., 2022). This trend has been reflected in a range of national and regional initiatives. For example, Fernandino Teens TV was introduced as a lecture-style educational television program to supplement printed modular instruction for junior high school students (Muñoz and Sanchez, 2023). In Indonesia, Muhammadiyah Sapen Elementary School rolled out Sapen-Radio and Sapen-TV during the pandemic and reported positive instructional outcomes (Nawangsari et al., 2022). Similarly, many countries and regions have launched television-based educational programs with local characteristics (Geni et al., 2021; Madhubhashini, 2021; Zeynivandnezhād and Naveedy, 2021). In some cases, educational television programs also add some interactive features to allow students to participate in classroom activities through the TV interface, so that the learning engagement is improved and the learning experience is better (Nwokedi et al., 2023). On the whole, these progress manifests that educational television is not only an emergency supplement to school education during the epidemic, but also a form of learning support, and its practical value is worth pondering. In the post-epidemic era, educational television may no longer be regarded as just a traditional media, but should be understood as a means of educational support that still works under certain circumstances.

Alongside this revival, the continued expansion of digital technology has not eliminated digital inequality; rather, it continues to reshape—in layered and often subtle ways—the opportunities students have to tap into and benefit from educational resources (Cheshmehzangi et al., 2023; Guo and Wan, 2022). Digitally disadvantaged students cannot be simply understood as those who are completely excluded from digital technology. They are actually students who are at a relative disadvantage in access and sustained use of digital learning resources (Martin et al., 2026; Van De Werfhorst et al., 2022). Such disadvantage can stem from a range of sources: tight household budgets, geographically lopsided distribution of educational infrastructure, weak digital skills, or even the fact that, in specific school or family environments, young people's use of personal devices is deliberately restricted (Martin et al., 2026). For some teenage students, learning with smartphones, tablets and computers is often subject to family supervision, school-related rules, or age-related management practices (Peltopuro, 2025; Pozsonyi et al., 2025). As a result, these students may not be completely unable to use digital learning, but in the daily learning process, they may still face practical difficulties in access and sustained use of mainstream digital learning tools (Gür and Türel, 2022; Pozsonyi et al., 2025). In this context, educational television programs can be used as a relatively accessible form of learning support, and in some cases, they provide a more inclusive option for students facing digital constraints (Jafar et al., 2023).

Against this background, it becomes important to ask whether adolescent students are inclined to turn to educational television programs in contexts where learning through personal digital devices is subject to restrictions, and to identify which factors influence their behavioral intention to do so. Compared with forms of online learning that rely primarily on smartphones, tablets, or computers, educational television may involve a relatively lower threshold for use in some contexts and may be more easily incorporated into everyday home-based learning routines (Gür and Türel, 2022). Accordingly, the present study adopts the UTAUT2 as its conceptual framework to investigate the key variables affecting digitally disadvantaged students' willing toward educational TV programming. (Venkatesh et al., 2012), with implications for the program design, the strengthening of support mechanisms, and the promotion of educational equity.

## 2 Literature review

### 2.1 Digitally disadvantaged students

Rapid societal digitalization has drawn increasing attention to digitally disadvantaged groups. In general, disadvantaged groups refer to individuals who are excluded from or have limited participation in economic, civic, and social participation. When the lens narrows to digitalization, what emerges is a disadvantage that manifests not merely as unequal possession of information and communication technologies (ICT) but also as unequal capacity to use these tools effectively and to draw genuine benefit from them. Previous studies have shown that individuals with disabilities, households facing economic hardship, and residents of underdeveloped areas are more likely to be tend to bear a heavier burden of digital inequality, because they often face structural barriers related to access, skills, and support (Arsenijevic et al., 2020; Pérez-Escolar and Canet, 2023; Tsatsou, 2022).

The term “digital divide” is most commonly understood as the gap separating people with scarce or no ICT access from those who enjoy plentiful access (Soomro et al., 2020). Early debates centered heavily on this access gap (Lythreatis et al., 2022). Later research moved beyond this perspective by emphasizing differences in technology use, digital skills, social support, and the actual benefits gained from technology use, leading to broader understandings of second- and third-level digital divides (Hoyos Muñoz and Cardona Valencia, 2025; Raihan et al., 2025; Robinson et al., 2018; van Deursen et al., 2016; Vassilakopoulou and Hustad, 2023). This shift suggests that digital disadvantage should not be understood simply as the absence of technology, but as a multidimensional condition involving unequal access, unequal use, and unequal outcomes (Liao et al., 2022). As technology continues its relentless march forward, the forms and scope of digital disadvantage continue to evolve (Aissaoui, 2022; Lythreatis et al., 2022).

Digitally disadvantaged students represent a specific subgroup within digitally disadvantaged populations in educational settings. Previous studies have shown that some students are placed at a relative disadvantage in digital learning because of limited economic resources, weaker educational and information infrastructures in their local contexts, or insufficient opportunities to access and use digital resources (Castaño-Muñoz et al., 2025; Katona and Gyonyoru, 2025; Robinson et al., 2018). Oyedemi and Mogano (2018) noted that, in South Africa, many students first encountered computers and the Internet after entering tertiary education, reflecting limited opportunities for digital access at earlier stages of education. Naidoo and Raju (2012) also used the term digitally disadvantaged students to refer to students who may require greater guidance and support in basic technology use and information-related learning contexts, a condition often associated with unequal educational opportunities and resource constraints.

In the present study, digitally disadvantaged students are defined as students who are in a relatively disadvantaged position as for access to and use of digital learning resources. This disadvantage is reflected not only in limited access to technology, but also in limited family economic resources, residence in rural or less developed areas, limited availability of personal digital devices, and practical obstacles to maintaining continuous use of digital learning materials. Accordingly, this study focuses on students whose digital learning conditions are relatively constrained and who may therefore depend more on alternative forms of digitally mediated learning support.

### 2.2 Educational television programs

Educational television has long been an important medium in the history of educational technology. Across a wide range of teaching and learning settings, television has served as a medium for instruction over many decades (Kocdar, 2017). Early studies suggested that television-based instruction could support student learning, reach large audiences at relatively low cost, and, in some cases deliver instructional content more effectively than conventional classroom teaching (Cain, 2017; Dolgova and Yu, 2020). With the widespread diffusion of television sets, educational television gradually became a practical channel for delivering learning resources to broad groups of learners. From a multimedia learning perspective, television provides combined visual and auditory input that can support learning and comprehension (Kuba et al., 2021) Additional studies have pointed to television's contribution in area-specific domains such as language development and language learning (Oktari et al., 2025; Peters and Webb, 2018). Taken together, these studies suggest that educational television has long had both instructional value and practical accessibility in educational settings.

For digitally disadvantaged students, the educational value of television is closely related to issues of access and inclusion. Compared with those learning formats that mainly rely on smartphones, tablets or personal computers, the threshold for acquisition and use of television-based learning may be lower. Earlier studies have found that poor economic conditions, unequal access to equipment, disparities in digital skills as core drivers of digital inequality (Lythreatis et al., 2022; Srivastava and Shainesh, 2015; Vassilakopoulou and Hustad, 2023). Against this background, educational television can be said to be an alternative form of learning support for those students with limited digital learning conditions. Some relevant studies have also mentioned that under the condition that the internet connectivity and digital devices are limited, TV-based learning can provide an easier and more practical support (Garcia-Aretio, 2022; Jordan et al., 2021; Santos and Santos, 2024). Therefore, educational television is not only a traditional instructional medium, but also a low-threshold support means that can help digitally disadvantaged learners. In educational technology research, the educational value of digital media depends not on its technical specifications alone but also on how it gets deployed in specific teaching and learning situations (Drijvers and Sinclair, 2024). Previous studies have also shown that digital technology's role in education is closely tied to the conditions of acquisition, the way of use and equity. The acquisition and using digital technology itself raises concerns worth attending to (Parks et al., 2025). For those students with relatively limited digital learning conditions, the significance of educational television is not only what teaching content it provides, but also that it can enter the daily learning environment with a relatively low access and use threshold, thus providing real support for learning.

Herein, educational television programs are defined as curriculum-related learning resources provided through television terminals and related television service platforms. This kind of program usually includes course instruction, knowledge support and other teaching resources aimed at primary and secondary school students. Compared with online learning forms that mainly rely on smartphones, tablets or personal computers, the threshold for access and use of educational television programs may be lower. This is especially targeted for those students with limited personal digital devices or insufficient stable digital learning conditions. For the group that this study focuses on, educational television is a real form of learning support that can be touched in a specific educational and technical situation, not just a continuation of traditional media. Therefore, examining students' willingness to implement educational television programs has a very clear contextual significance.

To grasp why students might-or might not-intend to use educational television programs, it is necessary to consider the factors that may shape such intention. Previous studies have pointed to education level, age, and income as pivotal socioeconomic determinants of digital engagement. Affordability of digital services, ownership of devices, and digital skills additionally shape how people access and use technology (Abid and Chaudhry, 2024). Other studies have further pointed out that the availability of technological devices and socio-economic status are important contributors to the digital divide (Mengstie and Sendek, 2023). However, these studies provide limited clarity regarding how these factors influence students' use intentions.

Although the existing literature has found some influential factors, how these factors affect the use intention of digitally disadvantaged students in different groups needs to be studied in depth. With the aim of systematically evaluating what drives the willingness of digitally disadvantaged students to utilize educational television broadcasts, this study borrows the theoretical framework of technology acceptance and use. Among the numerous technology acceptance frameworks available, UTAUT2stands out for its broad scope and strong applicability across contexts. And it provides us with a useful framework to analyze how different factors affect the behavioral intentions of digitally vulnerable students in using educational television programs.

## 3 Theoretical framework

### 3.1 UTAUT2

In this context, several influential models have been proposed, including the Technology Acceptance Model (TAM), Diffusion of Innovations (DOI), the Theory of Planned Behavior (TPB), and the UTAUT (Davis, 1989; Davis et al., 1989; Dearing and Cox, 2018; Kasprzyk, 2008; Rogers et al., 2014). These models have provided important theoretical foundations for examining technology acceptance from different perspectives (Williams et al., 2015). Notably, Venkatesh et al. (2012) provided the UTAUT, including behavioral intention and actual usage: PE, EE, SI, and FC. Crucially, these constructs do not dictate usage in a vacuum; rather, their influence on behavioral intention and ultimate behavior fluctuates depending on a user's age, gender, prior experience, and whether their technology adoption is voluntary. Compared with the earlier models, UTAUT demonstrated stronger explanatory power for user acceptance in the original empirical study (Tamilmani et al., 2021; Venkatesh et al., 2012). UTAUT added three constructs, namely, HM, PV and H, to gain a deeper nuance of explain the use of technology in the consumer context. Since most consumer behavior is voluntary, so the adjustment variable 'voluntariness of use' is removed, so as to get the UTAUT2. And it contains seven core constructs: PE, EE, SI, FC, HM, PV and H. In the consumer context, UTAUT2 explained 74 % of behavioral intention and 52 % of technology use (Venkatesh et al., 2016).

**Figure 1:**
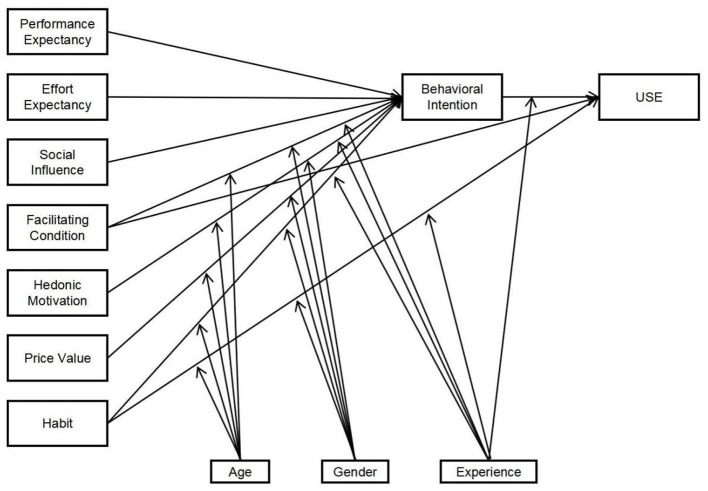
UTAUT2 model.

The UTAUT2 serves as the theoretical anchor for this research, enabling a systematic interrogation of the underlying determinants driving digitally marginalized students' willingness to adopt educational television programming. UTAUT2, a commonly utilized model in technology acceptance studies, explains individuals' intention to use technology through multiple constructs, including PE, EE, SI, FC, HM, PV, and H (Roy, 2025; Venkatesh et al., 2012). Herein, educational television programs are not only a form of educational technology, but also a learning support medium with distinct media characteristics. For students whose digital learning conditions are relatively constrained, educational television generally involves a lower threshold for access and use than learning formats that rely mainly on smartphones, tablets, or personal computers, making it particularly suitable for examining students' intention to use this form of learning support (Lythreatis et al., 2022; Vassilakopoulou and Hustad, 2023). In this context, students' willingness to use educational television programs may be related not only to their perceptions of learning effectiveness and ease of use, but also to family and school support conditions, media-related experiences, and pre-existing patterns of contact with the medium. In this sense, UTAUT2 is well suited to the analytical purpose of this study and provides an appropriate framework for evaluating what shapes the technology acceptance choices toward educational television broadcasts within digitally underserved student cohorts. Based on the UTAUT2 framework, the initial research model of this study is illustrated in Figure 1, which depicts the hypothesized relationships among the seven constructs and behavioral intention.

### 3.2 Research hypotheses

#### 3.2.1 Performance expectancy (PE)

PE captures the extent to which an individual thinks that employing a given technology will boost their job or task performance. Across a range of technology acceptance studies, learners perceive a technology as useful for improving learning effectiveness, they have a greater chance to become more determined about using it (Sergeeva et al., 2025). In the present study, if digitally disadvantaged students perceive educational television programs as helpful for understanding course content, enhancing learning effectiveness, or supporting academic performance, they are more apt to develop a favorable intention toward using these programs. Consequently, the hypothesis that follows is put forth:

***H1***. PE positively influences digitally disadvantaged students' behavioral intentions to adopt educational television programming.

#### 3.2.2 Effort expectancy (EE)

EE refers to the perceived ease associated with using a technology Research consistently indicates that technologies judged to be easier to operate enjoy higher acceptance rates (Nurlaela et al., 2025). Depending on the present study, if digitally disadvantaged students perceive educational television programs as easy to access, understand, and operate, it is more probable that they develop a stronger behavioral intention to use such programs.

***H2***. EE positively influences digitally disadvantaged students' behavioral intention to use educational television programs.

#### 3.2.3 Social Influence (SI)

SI denotes the degree to which an individual believes thatpeople who matter to them think they ought to adopt a particular technology. Scholarship has shown that social environments, including encouragement or expectations from parents, teachers, peers, and other significant groups, can influence users' intention to adopt technology (Garc'ia de Blanes Sebasti'an et al., 2025). In the present study, digitally disadvantaged students' willingness to use educational television programs may be shaped by the attitudes and suggestions of parents, teachers, and peers.

***H3***. SI positively influences digitally disadvantaged students' behavioral intention to use educational television programs.

#### 3.2.4 Facilitating conditions (FC)

FC capture the extent to which an individual believes that there are the tools and assistance needed to use a a technology. In educational technology contexts, access to equipment, technical support, and a supportive learning climate often affect users' readiness to take up a technology (Zhao et al., 2025). In the present study, if digitally disadvantaged students perceive sufficient technological and environmental support for using educational television programs, they are inclined to form a positive behavioral intention.

***H4***. FC positively influence digitally disadvantaged students' behavioral intention to use educational television programs.

#### 3.2.5 Hedonic motivation (HM)

HM pertains to the enjoyment or fun that a user derives from interacting with a technology. Prior studies have shown that when technology use is experienced as enjoyable, users tend to be more resolute in their intention to keep using it (Hu et al., 2025). In the present study, if digitally disadvantaged students perceive educational television programs as enjoyable or interesting to use for learning, they are expected to develop a stronger behavioral intention.

***H5***. HM has a positive impact on digitally disadvantaged students' behavioral intention to use educational television programs.

#### 3.2.6 Price value (PV)

PV concerns an individuals' assessment of the trade-off between a technology's alleged advantages and the costs related to using it. In technology acceptance research, users generally become more willing to adopt a technology when they believe that its benefits outweigh its monetary or time costs (Zhou and Chen, 2025). In the present study, the perceived costs associated with educational television programs may be particularly relevant to digitally disadvantaged students and their families. If they perceive these programs as worthwhile in relation to the required costs, they are more prone to form a stronger behavioral intention.

***H6***. PV positively influences digitally disadvantaged students' behavioral intention to use educational television programs.

#### 3.2.7 Habit (H)

H refers to the degree to which individuals perform behaviors automatically as a result of prior learning and repeated experience. Previous studies have shown that habitual use can strengthen users' intention to continue using a technology (Bessadok and Hersi, 2025). In the present study, if digitally disadvantaged students have already developed a tendency to use educational television programs as part of their learning routine, this habitual pattern is apt to reinforce their behavioral intention. The hypothesis is:

***H7***. H positively influences digitally disadvantaged students' behavioral intention to use educational television programs.

## 4 Methodology

### 4.1 Research design

Quantitative methods are appropriate for large-scale data collection and analysis and can yield statistically meaningful results. This research set out to untangle the drivers shaping how digitally disadvantaged students intend to adopt educational television programming. To gather standardized, quantifiable insights from a substantial participant pool, a survey questionnaire served as our primary methodological tool, with all latent constructs operationalized via a 7-point Likert scale anchoring from “strongly disagree” to “strongly agree.” The 7-point scale was adopted, mainly for two reasons. First, the original UTAUT2 scale developed by Venkatesh et al. (2012) uses a 7-point score. This study wants to be consistent with the existing measurement methods of this framework. Second, previous methodological and empirical studies have also mentioned that scales with more options can distinguish the judgments of respondents more carefully, and the score distribution may be more uniform (Dawes, 2008; Debets et al., 2020; Preston and Colman, 2000). Because this study examines the behavioral intentions of students using educational television programs, as well as related attitudes, perceptions, and evaluation constructs, the 7-point scale should be able to capture these differences more sensitively, and also furnish a solid basis for the PLS-SEM analysis that follow.

The study employed a cross-sectional design, meaning that all data were collected at a single time point. Cross-sectional approaches are practical for obtaining information from sizable samples within a relatively compressed timeframe and for providing a momentary snapshot of the phenomenon under examination (Aggarwal and Ranganathan, 2019; Setia, 2016).

### 4.2 Instrument and measures

To ensure construct validity, we tailored our data collection instrument based on the foundational metrics of the UTAUT2 model (Venkatesh et al., 2012) to measure students' behavioral intention to use educational television programs and the associated factors. To fit the context of educational television use examined in this study, several items were modified to reflect the particularities of the target group and the features of the medium to better reflect the specific use scenarios of educational television as a learning medium. Given that the respondents were adolescent students in a Chinese-speaking context, the original English items were first translated into Chinese, and the wording was then refined in light of the study context. Educational technology researchers on the research team reviewed the item wording to improve alignment between the questionnaire content and the research context. In addition, teachers and parent representatives reviewed the questionnaire to verify that the items were accurate, clear, and appropriate for adolescent respondents.

### 4.3 Sample and data collection

This study relied on a non-probability sampling approach. The data were collected in Zhejiang Province, China, through a mix of in-person paper-based questionnaires and online survey platforms. Finally, 376 valid responses were obtained. The students were between 12 and 16 years old. The reason for choosing this age group is that students at this stage are usually subject to family supervision and school management when learning with personal digital devices such as smartphones and tablets, and educational television is generally more accessible at home. In this way, this age group is very suitable for examining the behavioral intention of using educational TV programs.

The study's focus rests on young students who have relatively limited access to and sustained engagement with digital learning resources in daily learning situations. In this context, educational television can be regarded as a relatively easy form of learning support. In addition, Zhejiang 's own television coverage and user base are still quite large (National Bureau of Statistics of China, 2024), indicating that TV is still a practical medium in this region. This further shows that this sample is well matched with the research context. Among all respondents, 86.97% were female, and 37.96% reported that they had used or were currently using educational television programs in the referenced service context. This proportion reflects respondents' reported direct use within the referenced service context and does not necessarily indicate that the remaining respondents lacked general exposure to television as a medium.

Given that the respondents were mainly minors, questionnaire administration was organized primarily through schools, and some online responses were completed with parental assistance when necessary. Before the questionnaire was completed, teachers or parents briefed the students on the study's general purpose and the requirements for responding. During the response process, necessary clarification was provided when students had difficulty understanding particular items in order to support accurate responses. The study received approval from the relevant institutional ethics committee, and parents or guardians were informed of the survey arrangements in advance.

### 4.4 Data analysis

To examine the hypothesized relationships among the constructs, PLS-SEM was employed (Chin et al., 2003), which makes fewer assumptions about measurement scales, sample sizes, and data distributions than covariance-based methods (Seman et al., 2018). Path coefficient significance was evaluated through a bootstrapping procedure with 5,000 subsamples.

## 5 Results

### 5.1 Measurement analysis

Establishing the psychometric soundess of the structural model was an essential prerequisite before proceeding to structural paths estimation, ensuring our latent frameworks offered a dependable baseline. Consequently, three criteria guided this evaluation: internal consistency reliability, convergent validity, and discriminant validity. The resulting statistical outputs, including individual factor loadings, Cronbach's alpha, composite reliability (CR), and AVE, are delineated chronologically in Table 1. Overall, all factor loadings cleared the 0.70 benchmark, indicating that the indicators loaded satisfactorily on their intended constructs. The lowest loading was observed for SI1 (0.762), whereas the highest loading was found for HM2 (0.906), suggesting that all retained items made acceptable contributions to construct measurement. Cronbach's alpha values stretched from 0.718 to 0.862, while CR values ranged from 0.870 to 0.906, both pointing to acceptable-to-good internal consistency reliability across the board. Furthermore, the recorded AVE values comfortably surpassed the widely accepted 0.50 benchmark, spanning from 0.681 to 0.779, supporting convergent validity. Among the constructs, HM showed the highest AVE (0.779), indicating comparatively strong convergent validity, whereas H showed the lowest AVE (0.681), although this value remained well above the acceptable threshold.

**Table 1:** Loadings, AVE, composite reliability, and Cronbach's α.

Construct	Item	Factor	Cronbach's α	CR	AVE
Performance expectancy	**PE1:** Using educational TV programs allows me to complete my learning tasks faster	0.777	0.789	0.874	0.699
	**PE2:** Using educational TV programs can make it more likely that my grades will improve quickly	0.880			
	**PE3:** I think educational TV programs are generally useful	0.849			
Effort expectancy	**EE1:** Learning to use educational TV programs is easy for me	0.852	0.849	0.898	0.688
	**EE2:** I have a clear understanding of the function and use of educational TV programs	0.817			
	**EE3:** Learning through educational TV programs is simple	0.785			
	**EE4:** I can skillfully use educational TV programs for learning	0.862			
Social influence	**SI1:** My family suggested that I use educational TV programs	0.762	0.807	0.882	0.714
	**SI2:** Friends and classmates suggested that I use educational TV programs	0.900			
	**SI3:** Teachers and leaders advised me to use educational TV programs	0.867			
Facilitating conditions	**FC1:** My family has purchased or subscribed to educational TV programs	0.838	0.862	0.906	0.707
	**FC2:** I am able to independently acquire the knowledge required to use educational television programs (mainly referring to existing knowledge and skills, e.g., watching television, using the remote control, recognizing the Internet, etc.)	0.870			
	**FC3:** Educational TV programs and other digital learning apps I have used are compatible with each other, e.g., they all have the same practice problems, lecturers, etc.	0.841			
	**FC4:** When I have trouble with educational TV programs (cannot operate the device or do not know how to select other learning content), I can find professionals to help me	0.814			
Hedonic motivation	**HM1:** I enjoy using educational TV programs	0.858	0.718	0.876	0.779
	**HM2:** Using educational TV programs is interesting and enjoyable	0.906			
Price value	**PV1:** I think the current price of educational TV programs is acceptable to my family	0.864	0.815	0.890	0.729
	**PV2:** I think educational TV programs are currently affordable	0.871			
	**PV3:** For the current price, the educational TV programs provide good learning content	0.826			
Habit	**H1:** Using educational TV programs has become a habit for me	0.833	0.844	0.895	0.681
	**H2:** I have largely stopped using learning apps other than educational TV programs	0.811			
	**H3:** I can only use educational TV programs for learning at the moment	0.851			
	**H4:** Using educational TV programs for learning is the best option for me	0.805			
Behavioral intention	**BI1:** I plan to use educational TV programs for learning in the future	0.836	0.777	0.870	0.691
	**BI2:** I plan to continue to use educational TV programs frequently	0.828			
	**BI3:** I will always try to use educational TV programs in my daily life	0.830			

To assess discriminant validity, Table 2 presents the heterotrait-monotrait ratio (HTMT). All HTMT figures stayed within the commonly accepted bounds, signaling that the constructs were empirically separable. Regarding HTMT estimates, the peak correlation manifested between HM and BI, reaching a maximum of 0.618, followed by H and BI (0.596), suggesting relatively stronger associations for these construct pairs than for the others, while still within an acceptable range for discriminant validity.

**Table 2:** Discriminant validity: Heterotrait-Monotrait ratio (HTMT).

	BI	EE	FC	H	HM	PE	PV	SI
BI								
EE	0.303							
FC	0.468	0.284						
H	0.596	0.465	0.56					
HM	0.618	0.397	0.295	0.542				
PE	0.224	0.252	0.22	0.254	0.255			
PV	0.367	0.304	0.34	0.529	0.403	0.118		
SI	0.274	0.330	0.291	0.472	0.399	0.242	0.234	

Evidenced by the matrix in Table 3, the diagonal square root of the AVE for every individual latent dimension consistently exceeded its off-diagonal correlations with paired counterparts, thereby establishing discriminant validity via the Fornell-Larcker benchmark. Illustratively, BI yielded a square root AVE of 0.831, and a similar pattern was observed for the remaining variables. In sum, the measurement model assessment, summarized across Tables 1–3, demonstrated adequate reliability and validity, providing a sound basis for the structural model analysis that follows.

**Table 3:** Discriminant validity: Fornell-Larcker criterion.

	BI	EE	FC	H	HM	PE	PV	SI
BI	0.831							
EE	0.248	0.830						
FC	0.387	0.248	0.841					
H	0.486	0.396	0.481	0.825				
HM	0.467	0.308	0.241	0.426	0.882			
PE	0.180	0.213	0.188	0.212	0.203	0.836		
PV	0.295	0.256	0.294	0.440	0.311	0.100	0.854	
SI	0.233	0.273	0.250	0.394	0.310	0.208	0.190	0.845

### 5.2 Structural model analysis

**Figure 2:**
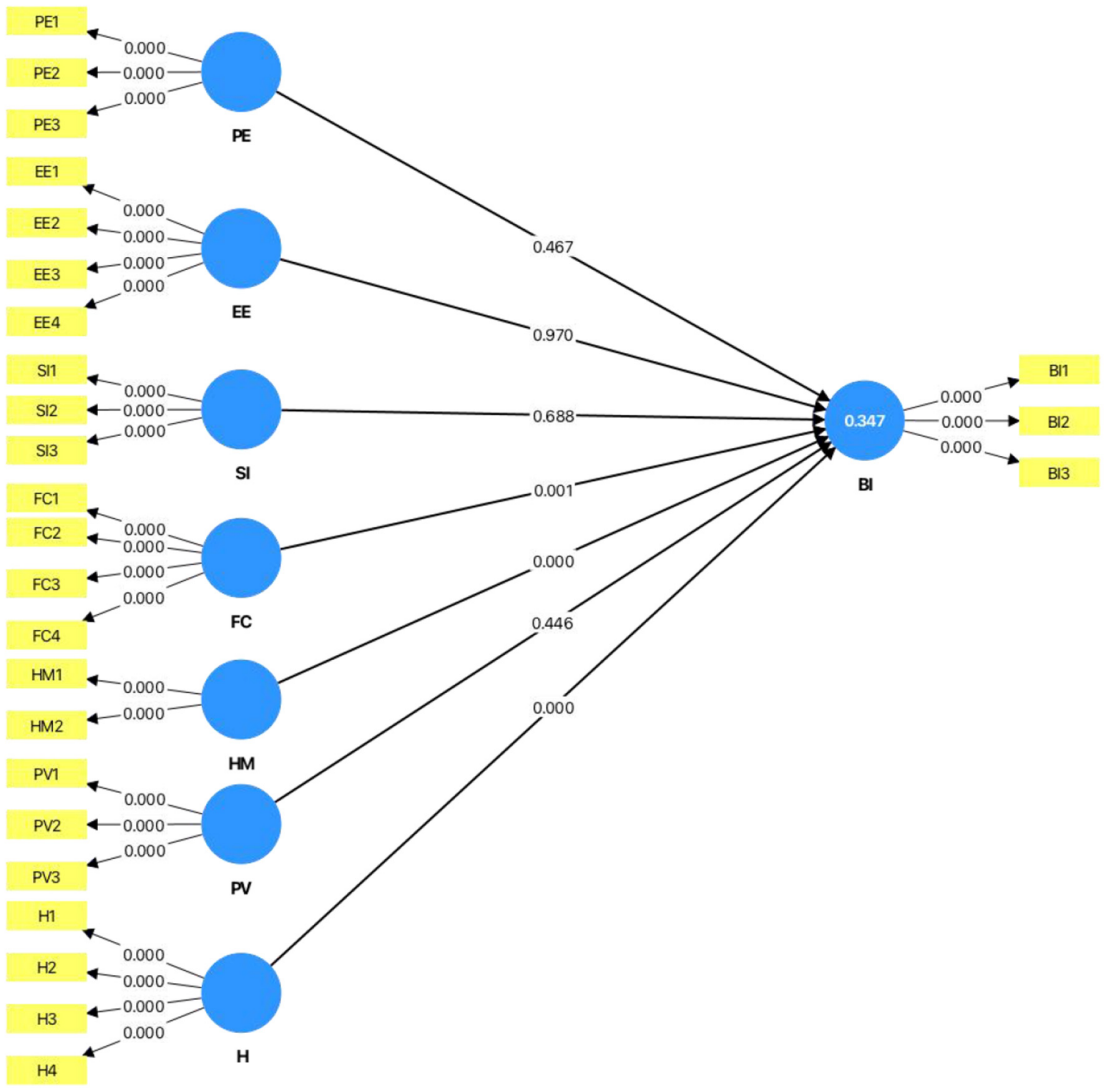
SmartPLS structural model output.

The structural model was assessed by inspecting the path coefficients (β), alongside their associated *t*-values and *p*-values, coefficient of determination (*R*^2^), effect sizes (*f*^2^), and the standardized root mean square residual (SRMR). Figure 2 presents the SmartPLS structural model output, and Table 4 summarizes the hypothesis testing results. The exogenous variables together accounted for 34.7% of the variance in behavioral intention (*R*^2^ = 0.347). As shown in Figure 2 and Table 4, three of the seven hypothesized paths were statistically significant. Specifically, HM exerted the strongest positive influence on BI (β = 0.304, *t* = 5.754, *p* < 0.001), followed by H (β = 0.256, *t* = 4.370, *p* < 0.001) and FC (β = 0.180, *t* = 3.441, *p* = 0.001). By contrast, the effects of PE (β = 0.031, *p* = 0.467), EE (β = −0.002, *p* = 0.970), SI (β = −0.020, *p* = 0.688), and PV (β = 0.037, *p* = 0.446) were all non-significant at conventional levels. With regard to effect sizes, HM posted the largest *f* ^2^ value (*f*^2^ = 0.107), followed by H (*f*^2^ = 0.055) and FC (*f*^2^ = 0.037), whereas the remaining predictors showed negligible effects. In addition, the SRMR value was 0.049, which falls under the commonly recommended threshold reported in prior research (Henseler et al., 2016).

**Table 4:** Hypotheses testing results.

Hypotheses	Path coefficient (β)	*t*-value	*p*-value	*f* ^2^	Results
H1:PE->BI	0.031	0.728	0.467	0.001	Unsupported
H2:EE->BI	−0.002	0.037	0.970	0.000	Unsupported
H3:SI->BI	−0.020	0.402	0.688	0.000	Unsupported
H4:FC->BI	0.180	3.441	0.001	0.037	Supported
H5:HM->BI	0.304	5.754	<0.001	0.107	Supported
H6:PV->BI	0.037	0.763	0.446	0.002	Unsupported
H7:H->BI	0.256	4.370	<0.001	0.055	Supported

Overall, the structural results showed three significant positive paths from HM, H, and FC to BI, whereas the remaining four hypothesized paths were not statistically significant.

### 5.3 Importance-performance map analysis (IPMA)

**Figure 3:**
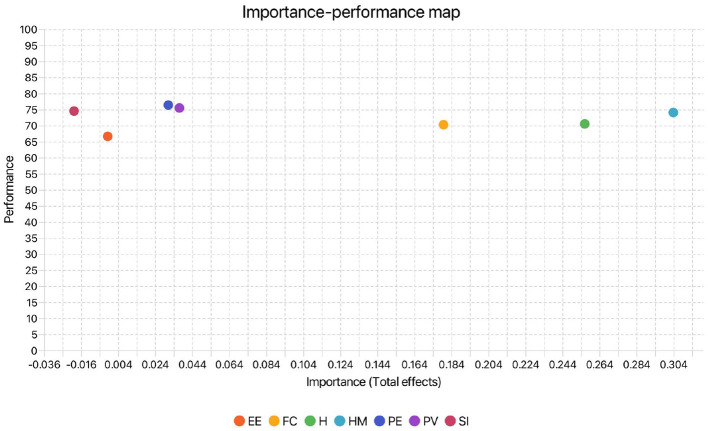
Results of IPMA.

IPMA was conducted to sharpen the interpretability of the PLS-SEM findings (Ringle and Sarstedt, 2016). Within the execution of the IPMA, Behavioral Intention was designated as the focal endogenous outcome variable. Figure 3 maps the results., HM emerged as the single most important predictor of digitally disadvantaged students' behavioral intention to use educational television programs, followed by H, FC, EE, SI, PE, and PV. In terms of performance, PE showed the highest score, followed by PV, SI, HM, FC, H, and EE.

## 6 Discussion

Grounded in the UTAUT2 framework, this inquiry scrutinized the underlying determinants driving the technology adoption tendencies of digitally disadvantaged learners toward educational television services. The empirical insights revealed a stratified impact among the predictors: enjoyment-driven impulses (HM) captured the greatest statistical weight, closely followed by the significant supportive roles of H and FC. Conversely, functional and contextual dimensions-specifically PE, EE, SI, and PV-failed to demonstrate critical predictive relevance. Taken together, the evidence paints a picture in which students' intentions in this particular educational niche are shaped more by media experience, daily contact, and actual support conditions rather than by instrumental evaluation, social norms or price considerations. Judging from the characteristics of this sample, educational TV is more like a supportive medium embedded in the daily learning at home, so the intention model used by students is somewhat different from the common report results of other digital learning technologies.

### 6.1 Main influential factors

HM stood out as the single strongest force behind digitally disadvantaged students' behavioral intention. This finding aligns broadly with prior educational technology research showing that enjoyment can be a decisive driver of learners' intentions (Meet et al., 2022). For the students examined in this study, the willingness to use educational television programs was influenced not only by the considerations of learning efficiency or functional value, but also by their sense of interest, enjoyment, and engagement during use. Educational technology research has increasingly emphasized that learning technologies should not only be functional and effective, but also engaging and enjoyable for users (Suhail et al., 2024). For educational television programs, this means that simply presenting instructional content may not be enough to strengthen students‘intention to use them, and that the program presentation and the viewing experience it generates can also affect students' acceptance of this learning medium (Muflihah, 2024). Practically, the design and implementation of educational television programs should attend not only to instructional content itself, but also to how the program is presented and experienced by learners in order to strengthen students' intention.

H likewise contributed a meaningful positive effect on behavioral intention. The literature has long noted that H can affect behavioral intention by strengthening individuals' daily contact and usage patterns of a medium or technology (Khemiri and Jallouli, 2025; Rashid, 2025). What this suggests is that students' intentions do not arise from a one-off evaluative judgment; they are embedded in ongoing learning engagement and pre-existing media habits. For those adolescent students who are subject to certain restrictions on learning with personal digital devices, educational television may be easier to integrate into home-based learning and become part of their daily learning support (González et al., 2022). Under such conditions, habit can not only enhance students' familiarity with educational television programs, but also strengthen their willingness to continue to use this medium. From a practical standpoint, the promotion and implementation of educational television programs should not only focus on the quality of the content, but also pay attention to the stability of students' everyday contact with the medium, so as to gradually strengthen habitual use and behavioral intention.

FC represented another important predictor of digitally disadvantaged students' behavioral intention to use educational television programs. Existing research has shown that access to necessary resources, support, and enabling conditions often affects individuals' acceptance of a particular technology or medium (Rudhumbu, 2022; Tseng et al., 2022). This indicates that although the threshold for access and use of educational television may be lower than some other forms of digital learning, whether to use it still depends on the actual support conditions. For the students who are concerned by the research institute, this kind of support may not only be reflected in the ability to access the media of television itself, but also in the home environment, whether the program content is available, the necessary technical help and practical assistance in the process of use. Educational television cannot function independently of supportive conditions; whether it can be effectively accessed and continuously used by students remains closely related to whether the surrounding environment provides sufficient access and practical support (Adhikari et al., 2023). Accordingly, the promotion and implementation of educational television programs should focus not only on optimizing program content itself, but also on the conditions that support students' actual access and use, such as resource availability, ease of access, and necessary support from families or schools, in order to strengthen students' intention to use them.

### 6.2 Non-influential factors and potential influential factors

Although several classic constructs in the UTAUT2 are often regarded as important predictors of behavioral intention, PE, EE, SI, and PV did not reach significance warrants some reflection. Yet in this study, digitally disadvantaged students' behavioral intention to use educational television programs did not entirely align with the instrumental, social, or price-related patterns commonly reported in studies of digital technology acceptance. A plausible interpretation is that, within the specific setting under investigation, these students did not treat educational television as a productivity-enhancing tool in the same way that users in other contexts treat productivity software or e-learning platforms.

Neither PE nor EE also turned out to be a significant predictor. This pattern is not fully consistent with some previous studies, as learners in many educational technology acceptance contexts often form intention based on whether a technology is perceived as useful for improving learning performance and easy to use (Parhamnia, 2022; Suhail et al., 2024). However, educational television is more like a supportive learning medium, rather than a typical digital platform where students will take the initiative to judge efficiency or operation complexity. For these students, first and foremost, the significance of educational television is that it is easy to obtain and can enter the learning context, not whether it is clearly regarded as an efficient or easy-to-use learning tool. A learning medium's acceptability may depend in large part on how well it is exposed to and utilized (Kumar and Owston, 2016). Therefore, merely promoting educational television programs by emphasizing the learning effect or easy operation may not be sufficient to significantly increase students' intention to use it, and it is more important to guarantee that such programs can really enter the learning situation in an accessible and acceptable way.

SI likewise had no discernible impact on digitally disadvantaged students' behavioral intention to use educational television programs. Although SI has often been identified as an important predictor of behavioral intention in many technology acceptance studies, particularly in contexts where individuals are unfamiliar with a technology or rely on external normative support (Gharaibeh et al., 2020; Gharrah and Aljaafreh, 2021; Nordhoff et al., 2020), this effect was not observed here. This may indicate that, for digitally disadvantaged students, willingness to use educational television programs depended more on direct contact with and actual experience of the medium than on the opinions or expectations of parents, teachers, or peers. Because educational television functioned primarily as a supportive medium embedded in home-based learning contexts, behavioral intention depended more on whether students had genuinely encountered, experienced, and accepted the medium than on external social encouragement or normative pressure. In practical terms, promoting educational television programs solely through encouragement from teachers, parents, or peers may not be sufficient to significantly strengthen students' intention to use them; creating opportunities for direct contact with the medium and positive experiences of using it may matter more.

PV also showed no discernible impact on digitally disadvantaged students' behavioral intention to use educational television programs. This pattern is not fully consistent with some previous studies, as individuals in certain technology acceptance contexts often form intention based on trade-offs between perceived cost and expected benefit (Chresentia and Suharto, 2020; Shaw and Sergueeva, 2019). In the context examined in the present study, however, educational television programs did not function entirely as a learning technology for which students made explicit price-based judgments. For these students, educational television was more likely to exist as a form of learning support already embedded in the household media environment. Their willingness to use it may therefore have depended less on subjective evaluations of price or cost than on actual experience, conditions of use, and perceptions of the content itself. When a learning medium is not directly perceived by students in cost-benefit terms, the predictive influence of PV on behavioral intention may be relatively limited. Practically, promoting educational television purely on the grounds of low cost is unlikely to be enough to meaningfully strengthen students' intentions improving students' actual perceptions of content quality and use experience may be more important.

## 7 Conclusion and future work

Based on the UTAUT2 model, this study investigated digitally disadvantaged students' behavioral intention toward educational television programming and the factors that underlie it. Our path analysis revealed a distinct polarization among the predictors: enjoyment-driven impulses (HM), routines (Habit), and structural support (FC) emerged as prominent positive catalysts for students' behavioral intention. In contrast, core utility and social dimensions-namely PE, EE, SI, and Price Value-exhibited statistically negligible predictive power. In conclusion, media experience, routine engagement and practical support conditions proved to be stronger determinants of students' intention than instrumental evaluations, social norms and price-related considerations. This investigation extends the application of UTAUT2 to the domain of educational television and to offers further evidence for understanding the acceptance of alternative learning media in specific educational settings. Educational television is a relatively unique learning medium compared to mainstream digital learning tools such as smartphones, tablets and computers. The constellation of factors that shape students' behavioral intention toward it may differ accordingly. The findings suggest that, in contexts of limited access to digital learning resources, educational television should be understood not only as a continuation of a traditional medium but also as a potentially accessible form of learning support.

Several limitations of the current study should be kept in view. First, the sample was drawn from Zhejiang Province in China and consisted mostly of respondents were between 12 and 16 years of age. Thus, the findings should be generalized to other age groups, regions and educational contexts with caution. Second, the study centered on behavioral intention rather than actual use, and not all respondents had direct experience with the referred educational television service context. Thus, the results are more appropriate to be interpreted as reflecting students' behavioral intention toward educational television programs in a concrete and recognizable reference context, rather than evaluations formed solely through sustained prior direct use. Therefore, the results should be interpreted mainly at the behavioral intention level and not be directly transferred to actual use behavior. Third, the digitally disadvantaged students discussed here were understood primarily as those who were limited in their access and sustained use of digital learning resources within a specific learning context. In future research, this context-sensitive understanding could be further elaborated by adding more specific background variables to identify and compare relevant groups in more detail. Finally, the study rested on cross-sectional, self-reported questionnaire data, and the results are thus mainly indicative of students' subjective perceptions and behavioral intention at one moment in time, without tracking how these perceptions or intentions shift over time.

Future studies could consider these results in a broader scope of regions, age groups, and educational settings, and include variables such as parental educational background, family media environment, and school support conditions. In addition, longitudinal designs, interviews, and mixed-method approaches can be utilized to explore students' exposure to educational television, use patterns, and behavioral intention development in greater detail.

## Data Availability

Data supporting this study cannot be made available due to the need to protect student privacy and confidentiality. Requests to access the datasets should be directed to Hong Hetiao at 20020383@hznu.edu.cn
